# UNDERSTANDING THE CARE NETWORKS OF INFORMAL CAREGIVERS OF SOUTH ASIANS WITH BREAST CANCER USING ATLAS CAREMAPS

**DOI:** 10.1093/geroni/igac059.2239

**Published:** 2022-12-20

**Authors:** Ranak Trivedi, Ambri Pukhraj, Shreya Desai, Akanksha Jain, Rashmi Risbud, Lidia Schapira, Dolores Gallagher-Thompson, Karl Lorenz

**Affiliations:** Stanford University, Palo Alto, California, United States; Stanford University, Stanford, California, United States; Veterans Affairs Palo Alto Health Care System, Menlo Park, California, United States; Veterans Affairs Palo alto Health Care System, Menlo Park, California, United States; University of California, Davis, Davis, California, United States; Stanford University, Stanford, California, United States; Stanford University, Stanford, California, United States; Veteran Affairs Palo Alto Health Care System, Stanford, California, United States

## Abstract

Breast cancer rates are increasing among individuals with a South Asian heritage, i.e., from India, Pakistan, Nepal, Bhutan, Sri Lanka, Maldives, and Bangladesh. Informal caregiving is vastly understudied among this population, despite being influenced by cultural mores such as collectivism, cancer-related stigma, and gender roles. The South Asian Family Approaches to Disease (SAFAD) study took a mixed-methods, observational approach to describe the care networks of informal caregivers via an adapted version of Atlas CareMaps. Thirteen caregivers (43.9+/-14.8y, 30.8% female) were interviewed and included 6 husbands, 1 wife, 2 daughters, 1 son, 1 brother, and 1 friend of the breast cancer survivors. Semi-structured interviews were designed to develop an adapted Atlas CareMap, a visual representation of the caregivers’ care network at the time of the interview. Atlas CareMaps depicted the number of people supported by caregivers, and who provide support; their relationship; the frequency, intensity, and type of care; and modes of communication used. Immediate or extended family members were the most common people included. Results indicated that: 1) caregivers reported 8.8□3.5 individuals in their care network, provided care to 3.5+/-1.8 individuals and received care from 7.3+/-3.5 individuals; 2) caregivers primarily received emotional support from others; 3) their survivors’ care teams were often included as a source of support and medical knowledge for survivors, but only three noted that the care teams supported them directly. Describing these networks is a key step to developing culturally-concordant programs that can support South Asian caregivers, even as they care for breast cancer survivors.

